# A scoping review on the barriers to and facilitators of health services utilisation related to refugee settlement in regional or rural areas of the host country

**DOI:** 10.1186/s12889-024-17694-9

**Published:** 2024-01-17

**Authors:** J. V. F. Coumans, S. Wark

**Affiliations:** School of Rural Medicine, University of New England, Armidale, NSW 2351 Australia

**Keywords:** Refugees/asylum seekers, Health services accessibility, Rural settlement, Barriers, Communication, Cultural awareness, Discrimination, Housing, Employment

## Abstract

**Background:**

Healthcare access and equity are human rights. Worldwide conflicts, violence, and persecution have increased the number of people from refugee or refugee-like backgrounds. Because urban areas are already densely populated, governments have aimed to increase refugee resettlement in rural and/or regional areas. Because of the complex healthcare needs of refugees, this creates challenges for healthcare service providers. Identifying barriers to accessing healthcare in rural areas is therefore important to better inform policy settings and programmes that will provide culturally appropriate patient-centred care to the refugee community.

**Methods:**

This review scoped 22 papers written in English between 2018 and July 2023 from five countries (Australia, New Zealand, Germany, Bangladesh, and Lebanon) in order to provide an overview of the barriers and possible solutions to facilitate refugees’ access to healthcare.

**Results:**

The reviewed literature summarised the perceptions of at least 3,561 different refugees and 259 rural health service providers and/or administrators and identified major challenges. These include communication (illiteracy in the resettlement country language and lack of a suitable interpreter), lack of cultural awareness of health services, discrimination, and access difficulties (transportation, availability of health specialist services, cost). As a consequence, it was identified that improving access to affordable housing, employment through credential recognition, competence-level education for children, facilitating language training, and adapting health information would increase resettlement and encourage access to healthcare.

**Conclusions:**

Refugees face significant barriers to accessing and engaging with healthcare services. This impacts their integration into rural communities and increases the prevalence of psychosocial issues like feelings of loneliness, low self-esteem, a lack of autonomy, and a lack of empowerment over informed decision-making, especially for women, jobless men, and the elderly. These findings support the need for additional support for refugees and healthcare providers to improve language proficiency and cultural competency. Policymakers need to improve the availability and accessibility of employment, housing accessibility, and service mobility. Additionally, more research is needed to assess the efficacy of emerging innovative programmes that aim to close the gap by delivering culturally appropriate patient-centred care to refugee communities in rural areas.

**Supplementary Information:**

The online version contains supplementary material available at 10.1186/s12889-024-17694-9.

## Background

The United Nations estimated that at the end of 2022 the total number of people from refugee or refugee-like backgrounds, i.e., people forced to flee their homes due to conflicts, violence, fear of persecution, or human rights violations, was 108.4 million people worldwide [1]. Following the 1951 establishment of the United Nations Convention relating to the Status of Refugees [2], the countries with the highest rates of receiving people from refugee or refugee-like backgrounds [hereafter referred to simply as refugees for simplicity of reading] requiring resettlement in 2022 were Canada (47,600), the United States (29,000), and Australia (17,300) [1]. According to the United Nations High Commissioner for Refugees (UNHCR), these host countries have a responsibility to provide appropriate support services to assist refugees both during and after their resettlement. However, it has become evident that, while host countries are generally supportive of resettlement, the actual host community within that country may be under-prepared or not receptive towards refugees, as settlement comes with a multitude of challenges that involve socio-cultural restructuring and reorientation [3].

As a consequence of the physical and psychological trauma refugees might have experienced — initially in their home countries, then during their transit to the host country and finally during the resettlement process — they may present with comparatively worse mental and physical health than the general population in which they settle [4–6]. Refugees may be granted free access to the local healthcare services to manage these issues, but the system’s capacity to manage their often-complex needs in a culturally, linguistically, and clinically adequate manner is challenging and often leads to further inequities in healthcare provision [7].

Historically, refugees have often been resettled into already densely populated urban areas, and few host countries have developed comprehensive policy and practice frameworks to support non-metropolitan alternatives [8]. One major impediment to rural resettlement is that healthcare access can be more difficult in a regional setting where both generalist and specialist medical and allied health services are not readily available. However, some host locations, including Australia, Canada, and Europe, have developed special programmes designed specifically for regional settlement [9–11]. This is the case in Sweden and Germany, for example, where they have dispersal policies to spread refugee populations outside of large urban centres [12].

Barriers to accessing quality healthcare services in urban areas have been reviewed previously and include language, cultural, and economic issues [5, 13]. As such, lack of family or social network, lack of entitlements, fear of losing employment or residency if affected by certain medical conditions, administrative hurdles, and communication barriers are common problems mentioned by refugees [13–18]. In rural and regional areas, these barriers can be even greater due to socioeconomic risk, affordability of care, and access to care [19]. So, identifying the specific challenges in accessing healthcare in these locations, and possible solutions, is of importance. To our knowledge, there are no published reviews that summarise the literature base regarding healthcare access specifically in rural and/or regional areas for refugees and asylum seekers. An initial and then updated (July 26, 2023) search of PROSPERO (https://www.crd.york.ac.uk/prospero/) also found no proposed or current systematic reviews relating to healthcare access for refugees in non-metropolitan areas.

This scoping review aims to address this gap by examining the literature regarding healthcare for refugees resettling in rural and regional areas. The over-arching goal is to gain clarity surrounding healthcare access issues associated with resettlement in rural and regional areas, identify potential gaps in service delivery models, and then use this information to suggest possible solutions to facilitate healthcare access for refugees.

## Methods

We used the Preferred Reporting Items for Scoping Reviews (PRISMA) guidelines and the following eligibility criteria:

- *Population:* Refugees who had resettled in a rural or regional area of a new country and/or healthcare professionals who engaged with refugees following resettlement in a rural or regional area. Studies that had refugees as a part of a heterogeneous population were included only if refugees were clearly identified as a sub-group within that study and if relevant findings were specifically discussed in relation to them as a distinct cohort;
- *Intervention:* Studies had to comment on refugee interactions with either a rural or regional health service or a health intervention within a rural area in the new country;
- *Outcomes:* Data on refugees' use of health-care services in rural and/or regional areas. Papers that discussed social determinants and interventions that could impact access to healthcare in these areas were also considered;
- Types of studies: All research methodologies, including qualitative, quantitative, and mixed methods, were considered for inclusion;
- Time frame and language: All research published within the past five years, from January 1, 2018 to July 2023, was considered. This range was chosen to ensure the findings are relevant to current practices

Exclusion criteria for this scoping review were:

- studies published in languages other than English;
- all reviews, conference abstracts, book chapters, letters to the editor, protocols, opinion pieces, commentaries and editorials; and
- any studies that did not focus on rural or regional areas but instead reported on metropolitan and high-density population areas.

Studies were identified through searches in four online databases (PubMed, Embase, Scopus, and Web of Sciences) for papers published up until July 2023. A research librarian assisted with developing the search strategy, and the following terms were set for the initial title and abstract review: “refugee*” OR “seeker*” AND “health service*” OR “hospital*” OR “community health*” OR “health system*” OR “health outcome*” OR “healthcare*” AND “regional*” OR “rural*” OR “provinc*”.

After searching the different databases, all identified studies were imported into Endnote software, and the duplicates were removed. The remaining papers were screened independently by the two researchers to remove irrelevant papers in line with the above exclusion criteria. The title and abstract of all remaining papers were then reviewed in line with the eligibility and exclusion criteria. The full text of the remaining studies was then reviewed to identify papers that were relevant. In the event of a disagreement regarding inclusion or exclusion, a discussion between the researchers occurred and consensus was reached.

The reference lists of these chosen articles were then hand-searched to identify any other potentially relevant articles. For each study selected, a table was generated to extract the primary data, including the author’s name, year of publication, funding information, country of resettlement, population studied, methodologies, aims, and main findings. As this is a scoping review, no formal evaluation of the quality of the papers was undertaken in order to ensure a broad range of research was examined.

## Results

### Summary of sample selection

**Fig. 1:**
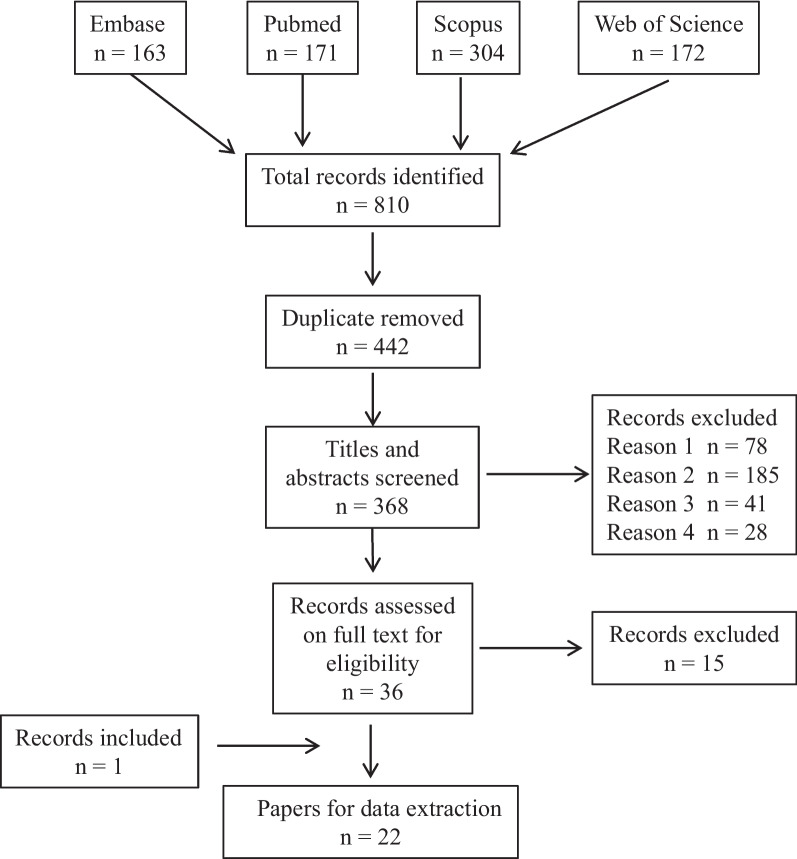
PRISMA Flow Diagram for the scoping review process

As can be seen in the following PRISMA flow diagram (Fig. 1), the initial search identified a total of 810 papers. After removing the duplicates (*n* = 442), 368 remained for initial screening for relevance. Of these 368 papers, a further 78 were excluded at this stage as they were reviews, conference abstracts, book chapters, letters to the editor, protocols, or editorials (Fig. 1, reason 1). An abstract screening of the 214 articles resulted in 180 additional papers being removed as they were either not focusing on healthcare barriers, social determinants, or interventions (Fig. 1, reason 2), refugees or asylum seekers (Fig. 1, reason 3), or issues associated with rural or regional settings (Fig. 1, reason 4). The full text of the remaining 36 papers was reviewed for inclusion in the study, and 15 were excluded based on our exclusion criteria. In addition to these 21 publications, one additional study [20], was identified through screening of reference lists. This resulted in a final sample of 22 papers.

### Sample demographics

A series of analyses examined a final sample of 22 papers (S1). Key demographic data included as a resettlement country: Australia (*n* = 17), New Zealand (*n* = 1), Germany (*n* = 1), Bangladesh (*n* = 1), and Lebanon (*n* = 2). This bias towards Australian research was considered by the authors; as noted in the Background, Europe and Canada also have specific protocols for rural resettlement, and it was therefore surprising that little research from those locations was identified. It is considered possible that the lack of published research from Europe and Canada may be the result of two issues; either a language issue, with papers published in French or Spanish, for example, or timing, whereby the findings from Europe and Canada simply have not been analysed and reported yet.

The perceptions of at least 3,561 different refugees and 259 rural health service providers and/or administrators were captured. Most studies looked at health access generally, although one study specifically examined the spatial accessibility of public dental services relative to the refugee population [21], while another reported on the major outcomes faced by mental health professionals working in the Rohingya humanitarian response [22]. Access to health services studied included all services, including local and governmental ones [20, 23–31], primary, secondary, and hospital [13, 22, 30, 32], mental [33, 34], mobile [35, 36], maternal and child health services [37], dental [21], online [25, 38, 39] and Médecins Sans Frontières [40].

Through this data extraction process, the major challenges associated with refugees and health service issues were identified, as well as possible facilitators and recommendations. These findings are summarised in Table 1 and fully described in the supplemental data (S1), and topics of confluence are discussed in greater detail below.

**Table 1:** Summary of key findings

Refugee/asylum seekers issues	Healthcare and context issues	Facilitators	Recommendations
Characteristics: • Communication or language difficulties • Low health literacy and healthcare familiarity • Poor education and digital literacy • Types of health issues • Non-transferability of qualifications Social/cultural: • Rely on connectivity and social cohesiveness • Fear of privacy breaches • Gender norms • Intergenerational tension • Non-transferability of qualifications • Employment delays • Discrimination and racism	Issues into practice: • Lack of adequate interpreters • Care delay or continuity • Inadequate cultural competency • Provider-patient power struggles • Limited number of GPs and specialists • Lack of cultural understanding leads to cultural and gender superiority • Treatment that does not take culture or beliefs into account • Reluctance to ask about the refugee or asylum seeker’s background Practical issues: • Lack of public transport • Housing affordability • Limited access to the social security system • Policy context and funding	Approaches to increase engagement: • Community-support, including religious groups, playgroups, and multicultural centres • Ties with real estate agents and landlords • People and community leaders from their home countries that live nearby • Employment or volunteering Service improvements: • Collaboration with migrant and refugee organisations and communities • Improving access to housing, social connections, education, and employment • Proximity of some services • Free or low-cost professionals	Possible approaches towards better care: • Creation language glossaries • Use dialect, images, audiovisual content, or phone calls from reputable service providers • Improve official government messages • Engaging community volunteers • Use of research as a medium for mutual learning and improving current services

### Communication

The most frequently mentioned topic was communication challenges [13, 20, 22, 24–35, 37–39]. Illiteracy in the resettlement country language is exacerbated by culturally inadequate health promotion programmes [26, 38, 39], and the lack of suitable interpreters, e.g., those who are remote, not of the same gender, or from a different cultural or religious group, often results in family members or children playing the role [13, 20, 27, 28, 30, 32–35, 37]. This causes problems such as care delays and continuity [13, 37]; fear of privacy breaches [13]; patient-provider power struggles, particularly for women patients who come from very patriarchal societies [13, 23, 26]; difficulties in obtaining informed consent [20]; and the inability to understand referral letters or telephone booking systems [24].

Language barriers also leads to difficulties understanding health issues, disease, and the healthcare system’s operation and navigation [13, 20, 24, 25, 28, 30, 32, 34, 40]. Specifically, for problems associated with accessing health-related information, it was identified that low proficiency in digital literacy is associated with lower education. This in turn leads to confusion and uncertainty in attempting to use of social networks to obtain information and support, and inhibits self-care practices, such as over-the-counter treatments and herbal supplements [24, 25, 34, 38–40]. This is a problem that is exacerbated if nurses are reluctant to ask about the refugees’ educational background, as the health professionals may lack sufficient detailed information from the referral process [37]. Additionally, social determinant problems associated with illiteracy were mentioned, which included: delayed employment [20, 27, 29, 32, 33]; difficulties engaging in the community, particularly for women; all of which lead to psychosocial issues such as depression, isolation, and loneliness [20, 26, 33].

### Cultural awareness

Refugees and health care providers also expressed concerns regarding the lack of culturally appropriate health promotion programmes, resources, and practises [22, 24, 26, 27, 30, 39]. For example, a lack of understanding among healthcare professionals about cultural, social, religious, and gender influences, as well as the impact of anxiety and trauma, causes distress, care difficulties, and delays, particularly with regard to mental illnesses, which are often perceived in terms of religious terms [34]. Refugees also reported perceived feelings of demonstrated cultural superiority from healthcare providers, while health professionals mentioned client passivity, particularly from women [23]. Within the broader community, refugees identified a lack of recognition of their knowledge, skills, and experiences. As a result, they reported low self-esteem. Refugees also expressed a desire to maintain their home language and cultural traditions, both of which cause intergenerational tension and stress in the family unit [27]. Collectively, these issues impacted the interpretation of important healthcare information due to the fact that medical treatments are predominantly delivered through a ‘western’ model [34].

### Discrimination

Another major source of concern is both perceived and actual discrimination from service providers and the broader community. Refugees linked feelings of discrimination to their need for individualised language support, as well as stereotypical views of cultural inferiority, which led to disrespect, disregard for personal choices, and inaction from health professionals [13, 20, 23, 27]. When refugees were not perceived as being ‘competition’ for resources and opportunities within the broader community, this feeling was partially alleviated by working or volunteering, as well as, for women, participation in playgroups. These activities contributed to the development of a positive self-image, daily routine and purpose, all of which aid the integration process and improve individual health status overall by encouraging healthy lifestyle behaviours and access to healthcare [22, 29, 37].

### Access to services

Access to culturally informed healthcare was identified as a significant problem in some situations. This is not surprising; as noted in the Background healthcare can be difficult to access in rural areas for all members of rural communities. Factors that were recognised as impacting on access included the size of the rural or regional town or city, a lack of transportation, the judgemental attitudes of some doctors, and the availability of interpreters and health specialist services [13, 21, 22, 30, 31, 33, 35, 37, 40]. In Germany, Hahn et al. [35] reported that the social security system can limit access to healthcare and specialists. Similarly, in Australia, the unaffordability of healthcare due to low income, inadequate funding support for refugees, and a scarcity of bulk billing GPs (e.g., the total cost of the medical visit is covered by the national Medicare benefit scheme) were also mentioned as barriers to access [25, 32].

### Possible facilitators

Community integration and, as a result, access to healthcare, are typically more difficult in rural areas where communities are often perceived to be ‘tight-knit’ and may be less welcoming of newcomers (i.e., long-term community residents are accustomed to knowing everyone and primarily interacting with friends and relatives, rather than with cultural ‘outsiders’), have fewer resources, and have fewer public transport options [41, 42]. Community integration necessitates a process of acculturation, e.g., a reciprocal adjustment and adaptation from the individual, as well as the development of community resilience in response to cultural diversity, all of which is affected by the perception of competing over tangible and intangible resources [3, 43, 44]. In addition, refugees often perceived themselves as outsiders due to language barriers, different cultural beliefs, and their unemployment status. As a consequence of these issues, it was identified that improving access to affordable housing within familiar communities, facilitating language support and industry-specific language training, employment access, and competence level education for children would be beneficial as they encourage healthy lifestyle behaviours and increase feelings of inclusion and belonging, all of which contribute to successful resettlement and encourage access to healthcare [27, 29, 31, 33]. Additionally, to facilitate communication, dissemination of health information, and prevention and self-management of chronic disease, the creation of flyers in different languages, the use of SMS messages, the adaptation of governmental messages to the different religious and cultural backgrounds, as well as the use of pictures and audio-visual messages with simple, clear, and concise language, will allow more refugees to be individually informed rather than having to rely on second-hand information from community leaders, friends, family, or staff from service agencies [24, 32, 36, 38, 39]. For mental health problems, the development of glossaries and cultural psychological measurement tools in the language of the origin country could be beneficial [22]. Also, because of the scarcity of highly skilled mental healthcare staff in some rural areas, the integration of mental health support into primary healthcare as well as engaging volunteers from the community to provide support and training healthcare providers to provide cultural safety could be considered [22, 24, 26, 30, 31]. In that regard, refugees with previous qualifications also mentioned a desire to join the healthcare system, but unfortunately, this is impaired due to a lack of credential recognition and potential institutional racism [31].

## Discussion

This scoping review highlighted the fact that healthcare barriers that impact the integration of refugees into rural or regional areas are multidimensional and subject to variations in locally-specific processes. This scoping review identified challenges around effective communication, arising from problems such as a lack of interpreters and language barriers resulting from poor literacy, as a prominent barrier for refugees to access healthcare [13, 15, 45, 46]. As previously identified, this difficulty arises from the fact that refugees may have minimal literacy in the settlement country’s language and that there is a lack of appropriate interpreters and flexible support to learn that language, which gender responsibilities further exacerbate. Language barriers cause issues associated with access to health care services, continuity of care, differences in interpretations, but also fear of privacy breaches, delays in employment, and discrimination [13, 15, 47]. Refugees are already a high-risk group for both physical [48] and mental [49] health problems, and those chosen for regional settlement may lack pre-existing social or family ties in the resettlement area, which is a key factor for integration [50]. These factors result in a higher prevalence of psychosocial issues like feelings of isolation, loneliness, low self-esteem, a lack of autonomy, and empowerment over informed decision-making [20]. It is acknowledged that many refugees will have experienced significant psychological pressures (i.e., unsafe routes, high uncertainty, living in camps, etc.) and that this could result in emerging and ongoing mental distress and psychopathology, including anxiety, depression, and post-traumatic stress disorder (PTSD) [51].

Another issue that is commonly identified in the literature is the requirement for an increase in the number of medical professionals who have appropriate cultural competence. The analyses reveal that there is a lack of understanding of gender boundaries in the discussion of sensitive topics and self-advocacy issues [26, 52] and differences in illness perception due to disparities in religious beliefs and practices [22, 24, 34], all of which result in difficulties in understanding health information and treatments [38, 39]. All of these factors make it more difficult to engage with and navigate the healthcare system, particularly for women, and can lead to care inequities [53]. These issues can be addressed by increasing cultural competency and safety among health professionals through targeted education and training, organisational policies and practices, and broader structural reform [54]. Also, health professionals should be trained to reflect on their power, the privileges associated with their own culture, and their cultural beliefs. If health professionals are trained to do so, it may assist in reducing the feelings of discrimination [20, 52] and ‘microaggressions’ [23] that refugees experience from health professionals. Instead of supporting engagement and facilitating individuals to have an active role in maintaining their personal health, these issues impact the ability of healthcare professionals to provide patient-centred care [55] and impair the therapeutic alliance [56].

A number of sub-groups were identified as being at higher risk. Women, men who are jobless, and the elderly experienced more difficulties integrating into the community, putting them at greater risk of experiencing mental problems [20, 23, 26, 33]. In the case of women, this is due in part to the global pre-existing gender inequities, such as being unpaid or undervalued workers, that still affect women [57]. A recent scoping review that investigated how gender affected refugee health during resettlement generally in Canada found that many refugee women are impacted by gender roles and expectations, as well as exposed to gendered health systems and practices that are harmful to health, especially mental health and access to services. They suggested that in order to mitigate the detrimental effects of gendered beliefs and practices on health during resettlement, resilience and community building are required [58].

### Differences between a rural or regional location and urban areas

As previously identified from studies carried out in urban areas [13, 15, 46], there are universal barriers to accessing healthcare services, such as low language proficiency resulting in a feeling of discrimination, poor knowledge of the country’s healthcare system, and a lack of cultural competency among healthcare professionals. However, these issues are greatly accentuated in rural areas, which also have unique challenges such as reduced public transport facilities, a lack of primary and specialist healthcare professionals, and low employment opportunities. It is recognised that people living in rural areas experience significant shortfalls in health services access [59], which is further exacerbated by physicians’ personal and professional goals being unfulfilled, as well as a lack of sense of belonging in the communities [60, 61]. Unfortunately, when combined with underemployment and rising healthcare costs, this affects access to appropriate healthcare and adds to the mental stress already experienced by refugees [62].

These findings support the need for increased targeted governmental interventions to better support health professionals and refugees before increasing regional resettlement. For example, to overcome the language barrier, digital communication assistance tools could be developed and used, as that has been shown to be a feasible approach to obtaining medical histories from foreign-language patients [63]. To alleviate the difficulties around transport, initiatives such as the University of Iowa Mobile Clinic, which is run by an interdisciplinary team of students under the supervision of physician faculty advisors, could provide additional basic services in rural communities [64]. In rural and remote communities where health students are not freely available, other health workers, such as nurse practitioners or allied health professionals, could eventually provide these basic services.

### Study limitations

As previously identified, the major limitation is that the majority of the papers found in the 5-year search period originated from Australia, where the government has been actively working to increase settlement in rural or regional regions [9]. Therefore, the findings of this scoping review may be limited in their applicability to other countries where healthcare services may be distributed differently, depending on the size of the rural or regional towns, government support, and cultural norms in place [65, 66]. Also, most of the studies are of a qualitative nature, with discrepancies between the refugee groups studied and local conditions. As such, the findings’ applicability might need to be interpreted and adapted by readers. Finally, as this is a scoping review, the search was restricted to scientific papers published in English, used broad inclusion and exclusion criteria, and didn’t conduct a quality assessment of the reviewed studies. Thus, any interpretation of the results should be made within that context.

## Conclusion

Facilitating and improving access to healthcare for refugees in rural areas is multidimensional, requiring additional support for both the refugees and healthcare providers. For refugees, particularly women and the elderly, the development of easily accessible language education and health literacy programmes and facilitated access to community groups, volunteering, and employment possibilities would be beneficial. For service providers, while education towards delivering appropriate culturally sensitive care for this vulnerable population already occurs through lectures and discussions, application of material learned through, for example, cultural immersion is less common, and this could help overcome practise shortcomings [67, 68]. At the government level, policies need to be adapted in terms of employment, housing accessibility, and service mobility. Finally, more research from a much broader range of locations and policy settings is needed to inform the development of innovative programmes. It is also clear that there is a lack of carefully designed evaluation programs regarding the effectiveness of delivering culturally appropriate patient-centred care for the refugee community in rural locations.

### Supplementary Information

**Additional file 1: Supplemental data 1 (S1).** Summary of data’s papers included in this scoping review.

## Data Availability

All data generated or analysed during this study are included in this published review and its supplementary information files.
