# Supplementary material for: A scoping review on the barriers to and facilitators of health services utilisation related to refugee settlement in regional or rural areas of the host country
Source: BMC Public Health. 2024 Jan 17;24:199. doi: 10.1186/s12889-024-17694-9 (PMC10792843; doi:10.1186/s12889-024-17694-9)
Supplement: Supplementary file 1 — Additional file 1: Supplemental data 1 (S1). Summary of data’s papers included in this scoping review. [file 12889_2024_17694_MOESM1_ESM.docx]

Supplemental data 1 (S1): Summary of data’s papers included in this scoping review.

| **Authors and funding** | **Country of resettlement** | **Population, country of origin (if know) and year of data collection, healthcare service used.** | **Research design (1), data collection (2) and analysis method (3)** | **Aims** | **Main findings** |
| --- | --- | --- | --- | --- | --- |
| Au et al. (2021)  Partially funded by James Cook University | Northern Queensland, Australia | Refugees (n = 14) from Iraq (n = 1), Congo (n = 6), Burma (n = 4), and Somalia (n = 3)  Late-2018 and mid-2019  Primary and hospital | (1) Qualitative  (2) Semi-structured interviews  (3) Thematic analysis | Examine refugee perceptions of healthcare in a regional centre (Townsville). | Perceived and experienced discrimination that might be due to language support needs.  Delays in care result in clinically adverse events, with blame shifting to institutional structural issues (lack of interpreters).  Fear of privacy breaches, which could result in stigma in the community. A fear more prevalent in women.  Language, the need for an interpreter, and the fear of privacy breaches were significant barriers affecting their understanding of health, disease, and health system navigation.  Refugees spoke positively about living in a regional city because of the proximity of some services compared to metropolitan cities.  Refugees from countries with large provider-patient power differentials struggled to advocate for themselves. |
| Bourke et al. (2019)  No information on funding | Victoria, Australia | Refugees and asylum seekers in a regional centre (n = 31)  Sudan (n = 6)  Congo (n = 7)  Iraq (n = 8)  Afghanistan (n = 10)  Healthcare services | (1) Qualitative  (2) Semi-structured interviews  (3) Thematic analysis | Explore the role of culture in shaping human experiences related to health. | use of interpreters who spoke a different dialect or who were not proficient in their language. Lack of understanding regarding standard protocols and processes operating within the healthcare system. Women spoke of feeling "stuck at home" and socially isolated, a situation accentuated by the language barrier. Jobless men reported a similar feeling. Inability to connect with local ‘Australian’ communities had psychosocial impacts (depression, isolation, and loneliness). Experience disrespect and disregard for personal and cultural choices. |
| Davison et al. (2023)  No funding | Regional Australia | General practitioners (GPs) (n = 30)  September to November 2020  Primary care | (1) Qualitative  (2) Semi-structured interviews | Explore GPs' perceptions of the challenges and facilitators of managing refugee healthcare needs. | Challenges to communicating with patients due to the very limited number of telephone interpreter services and the fact that interpreters speak a different dialect, as well as scheduling calls. Cultural differences and a lack of cultural familiarity. Sensitivities surrounding gender, lack of health literacy, and healthcare familiarity. Strong gender preferences for treatment and translator.  A limited number of GPs in the area is associated with longer consultations, adding significant financial costs to local practices.  Lack of local specialists and limited government-provided services.  Prevalence of trauma and somatic symptoms.  Lack of refugee health nurses.  Benefit of community support to facilitate healthcare service provision. |
| Elshazly et al. (2019)  No financial support | Cox’s Bazar District in south-east Bangladesh | Rohingya refugees  Basic, primary, secondary, and hospital | (1) Qualitative  (2) Conversations among the authors are all mental health professionals with working experience in the Rohingya humanitarian response and work for different organisations. | Discuss the most pressing mental health and psychosocial support (MHPSS) challenges in Bangladesh's Rohingya crisis and reflect on how to increase MHPSS assistance to the Rohingya population. | Language barriers: 62% of the survey sample were unable to communicate with the humanitarian services’ providers.  Because mental health issues are often perceived in religious terms, people only seek medical help when they are associated with physical symptoms.  Coordination of mental health and psychosocial support services.  Human resources are difficult to maintain.  Monsoon-related events increased stress, potential disasters, and the probability of service interruptions.  Inadequate psychological measurement tools, requiring a cultural adaptation of psychological measurement tools.  A lack of capacity to manage people with severe or complex mental health issues.  Limited staff care or support programmes with the possibility to foster a positive institutional climate with open attitudes towards stress and wellbeing.  Tensions between refugee and host communities are due to environmental degradation and competition between local and refugee populations over resources, opportunities, and cultural choices.  Proposed solutions:  Creation of Rohingya language glossaries with mental health terms for workers.  Engaging community volunteers and traditional and religious healers to provide support.  Integration of mental health support into primary health care and within health protection interventions. |
| Haderer et al. (2021).  Funding provided by Médecins Sans Frontières, Operational Centre Belgium, Brussels, Belgium. | Rural setting in Lebanon (two sites, Wadi Khaled or Shatila) | Syrian refugees with diabetes mellitus type 1 or type 2 (n = 29) who visit Médecins Sans Frontières clinics.  February and March 2019  Médecins Sans Frontières (MSF) cliniques | (1) Qualitative  (2) In-depth interviews  (3) Thematic analysis using Nvivo software. | Explore factors influencing the choice of and pathways to diabetes care. | Subjective knowledge of diabetes and care influenced the type of care provider chosen. For those who did not understand the value of routine, getting help was put off until the development of acute symptoms or a worsening of their condition.  Family and peer networks, perceived equality, distance, economic factors (direct consultation fees, drug purchases, diagnostic tests, etc.), and their legal status all have an impact on the choice of care provider.  Seek care from different ‘combinations’ of health-care providers.  Often, pharmacies serve as the principal provider and only use a clinician in the case of acute symptoms.  A lack of orientation in health-care services in Lebanon on arrival results in Syrian refugees still relying on their social networks to obtain their medication from Syria before accessing the Lebanese health system. |
| Hahn et al. (2020)  Funded by the Damp Stiftung | Rural area in the federal state of Schleswig-Holstein, Germany | Refugees living in refugee accommodation in rural areas (n = 12), health professionals (n = 7), and administrators (n = 6).  Refugees from Iraq (n = 7) and Afghanistan (n = 5)  July 2016 to February 2017.  Mobile Medical Practice | (1) Qualitative  (2) Semi-structured interviews  (3) Qualitative content analysis | Explore the experiences of refugees, health care professionals, and administrators involved in refugee health care. | Limited access to the social security system is a barrier to accessing health care.  The Asylum Seekers’ Benefits Act restricts access to health care.  Lack of public transport and the availability of health care staff in rural areas.  Permission from the social welfare office to see a specialist.  The availability of interpreters limited access to health care. |
| Hawkes et al. (2021)  No external funding. | Tasmania, Australia | Women of refugee background (n = 9) and service providers (n = 12).  May 2019 to August 2020  Mainstream Mental Health Services | (1) Qualitative  (2) Semi-structured interviews  (3) Thematic analysis | Explore the resettlement stressors for women of refugee background. | Communication, a lack of fluency in English to express needs, difficulties attending language classes as women are the primary children’s caregivers, and never participating in an educational setting led to a lack of opportunities to form relationships in new communities.  Challenges in accessing everyday basic needs in particular accommodations.  Dependence on government support and difficulty finding paid employment forced refugees to relocate from regional to metropolitan locations.  Loss of connection to the culture of origin and concerns associated with children developing a connection to the Australian culture create intergenerational tension and stress in the family unit.  Inability to access mainstream mental health services for help.  Lack of interpreters and the fact that regional locations are under-resourced and under-funded. |
| Healey et al. (2022, (a))  Funded by the Hunter New England Area Health Service. | Hunter New England region, NSW, Australia | Afghan (n = 5), Congolese (n = 6) and Syrian (n = 4) refugees who arrived in Australia on or after 1/1/2014.  COVID-19 information | (1) Qualitative  (2) Semi-structured interviews  (3) Thematic analysis  Interviews, March to August 2021. | Explore how culture, refugee experiences, and existing relationships shaped what COVID-19 messages were listened to and shared. | Due to inadequate schooling, limited English proficiency, and diverse abilities in digital literacy, information literacy, and media literacy, being a refugee influenced access to COVID-19 health messages, which in turn led to low self-confidence.  Anxiety and trauma-related mental health issues affected how refugees interpreted and responded to crucial information.  Place trust in local leaders, English-speaking or educated family members and friends, as well as agency workers.  Rely on connectivity and social cohesiveness to easily communicate knowledge in their native tongue.  The multiplicity of languages, religions, and cultural backgrounds was poorly addressed in official COVID-19 communications.  Prefer simple audio-visual COVID-19 messages that could be accessed through smartphones (social media, text messages, phone calls, emails, government websites, and applications).  There is a need to improve official government messages to reach those with basic education levels.  They recommended enhancing the role and response of Refugee Health and other public health organisations.  Use of research as a medium for mutual learning and improving current services. |
| Healey et al. (2022, (b))  Funded by the Hunter New England Area Health Service | Armidale, NSW, Australia. | Ezidi (or Yazidi) refugees (n = 600) are an ancient ethnic minority community that is indigenous to parts of Iraq, Syria, and Turkey. Many are from rural and impoverished backgrounds.  May 2021.  COVID-19 information | (1) Qualitative  (2) Semi-structured interviews  (3) Thematic analysis | Explore within the Ezidi refugee community how COVID-19 information has been communicated and shared. | Low literacy in English and Kurdish-Kurmanji, as well as in the usage of technology equipment, hinders the transmission of COVID-19 messages.  Underlying mental health issues related to the refugee experience have profound effects on memory and retention.  Cultural, socioeconomic, and gender factors all have an impact on the use of COVID-19 information, with older men and women facing greater difficulties. As a result, there is a reliance on trusted individuals (family and friends in the community and overseas) and service providers (schools, tertiary education, settlement services, and refugee health nurses).  The available government COVID-19 information was inconsistent and contributed to community confusion.  Use of Kurdish-Kurmanji dialect, images, audiovisual content, or phone calls from reputable service providers who need to deliver a straightforward, succinct, and pertinent message to improve COVID-19 communication. |
| Mohamed Shaburdin et al. (2022)  Funding from the Australian Research Council and support from the Australian Government Department of Health Rural Health Multidisciplinary Training Programme. | Rural Victoria, Australia | Staff (n = 21) from two small rural government funded health services located in significant culturally diverse locations.  November 2017 to April 2018.  Government funded health services. | (1) Qualitative deconstructivist approach  (2) Semi-structured interviews  (3) Thematic analysis. | Explore how staff construct ‘truths’ or racialised discourses about service users who are of different cultural backgrounds. | Reported on three types of microaggressions:  Criticise the refugee cultures that prevent them from engaging with them and imply that they are not to blame instead of adjusting their model of care to be inclusive and accessible.  There is evidence that health professionals assert cultural superiority and speak with strong racial overtones by expressing the cultural inferiority of certain groups.  Mention how some people talk about how women from immigrant and refugee backgrounds need to be "empowered."  Health professionals shared stories that imply passivity, discomfort, and, at times, relegating the ‘difficult’ client to a culturally-specific specialist service. This suggests that there is a reluctance among health professionals to self-reflect on the way they talk about particular service users. |
| Mwanri et al. (2023)  A small amount of internal funding from Flinders University,  South Australia. | Rural town South-Australia | Refugees (n = 44)  from Africa and Southeast Asia. | (1) Qualitative  (2) Semi-structured interviews  (3) Thematic analysis. | Explore facilitators and  barriers to successful resettlement in a rural setting. | Importance of community connections to facilitate settlement.  Importance of understanding the language and connecting with their faith.  Benefits of bridging ties with real estate agents and landlords.  Language barriers are a major challenge to accessing healthcare, which is exacerbated by the lack of interpreters in the right dialect.  Difficulties in accessing services and specialists are often located in larger cities.  Motivation to join the healthcare workforce but experience difficulties due to a lack of recognition and racism. |
| Nunn et al. (2022)  Funding from the Manchester Metropolitan University – La Trobe University Collaborative Project Grant Scheme, 2019. | Bendigo, Victoria, Australia | Karen refugees are a religiously and linguistically diverse group whose homelands are in the mountainous border zone between Myanmar, Thailand, and Laos (n = 52).  Late 2019  Local healthcare service | (1) Qualitative  (2) Community-based participatory research.  (3) Thematic analysis | Inform on the development and implementation of the research and share the findings in the health sector. | Physically: no pain, robust and energising feelings, and a "fresh" appearance with unblemished skin and eyes.  Social: having the capacity to engage in daily activities (communicating, going out with friends, contributing to the community, driving, playing sports, cooking, cleaning, and going to work and school).  Emotional: a comparatively low level of anxiety and tension, optimism about the future, maintaining a positive outlook, and being honest with people about your feelings.  A lack of sense of belonging, difficulty understanding referral letters or telephone booking systems, or trouble locating services in unfamiliar areas of the city or large buildings are all indicators of communication problems, especially for older people.  Prefer to rely on over-the-counter remedies, self-care, drugs, and herbs from the country of origin, as well as religious practises, and only seek professional medical help for severe or persistent problems.  Use the community as the most important source of guidance, support, and care. Request that healthcare professionals practise cultural safety. |
| Robards et al. (2019)  Funded by the NSW Ministry of Health. | Rural and regional NSW, Australia | Young people from marginalised groups, including refugees.  Refugees (n = 9) within marginalised groups (n = 41).  March 2016 to May 2017.  Any service delivering healthcare, including online services. | (1) Qualitative  (2) Semi-structured interviews  (3) Nvivo software and grounded theory | Understand health system navigation, including the use of technology, for young marginalised people, including refugees. | Select healthcare providers they identified as allies through trusted governments’ and organisations’ websites.  Convenience, engagement, the ability to provide holistic therapy, and affordability all play a role in healthcare journeys.  Discrimination results in forgone care, especially for refugees who face it because of their cultural background, level of language proficiency, and gender.  Being part of a marginalised group makes it difficult to navigate the health system.  Difficulties in understanding how to access the range of services they require. This varied according to the level of family support and autonomy.  Appreciated free or low-cost professionals and community-based youth workers. |
| Saleh et al. (2018)  No funding information | Rural areas and Palestinian refugee camps across Lebanon | Primary health care centres (n = 16).  Controls (n = 8, including 926 patients).  Interventions (n = 8, including 1433 patients).  June 2015 to June 2016.  Mobile health | (1) Quantitative. (2) Quality indicators from patients' records for diabetes mellitus and hypertension.  (3) Descriptive analysis of baseline characteristics of participants, bivariate analysis, logistic, and linear.  regression using SPSS. | Assess the effect of employing low-cost mHealth tools on the accessibility to health services and improvement of health indicators for individuals with noncommunicable diseases. | Weekly SMS messages aimed at the prevention and self-management of hypertension and diabetes enhanced BP control and decreased HbA1c levels.  The mHealth programme isn’t supporting lifestyle behaviour changes such as smoking cessation.  Targeted SMS reminders had no discernible impact on patients' use of primary care services or their visits for HbA1c testing, eye exams, or foot exams. |
| Shrestha-Ranjit et al. (2020)  No information on funding | Two regional towns of New Zealand | Refugee from Bhutan (n = 40).  Health professional (n = 12)  Data collected in 2014.  Healthcare services | (1) Quantitative longitudinal study.  (2) Individual interview.  (3) Thematic analysis | Examine the accessibility and acceptability of health promotion services in New Zealand for minority Bhutanese refugee women. | Findings from refugees  Illiteracy in English for women is a barrier to progression.  Lack of recognition of knowledge, skills, and experiences leads to difficulties in finding a job matching their skills.  Lack of involvement in decision-making activities such as community meetings.  Lack of culturally and linguistically appropriate health promotion services could be alleviated by hiring skilled Bhutanese people from the community.  Findings from health professionals  There are significant knowledge gaps about their health and medical treatments because they do not ask any questions or complain.  Lack of culturally and linguistically appropriate health promotion services and/or resources. |
| Smith et al. (2019)  In‐kind support through the Centre for Rural Health at the University of Tasmania. | Regional Launceston, Tasmania, Australia | Adult and youth community members and group leaders from Afghanistan, Bhutan, Burma, Sierra Leone, Sudan, and Iran (n = 31).  Mental health and support services. | (1) Qualitative  (2) Semi-structured interviews  (3) Thematic analysis using Nvivo software. | Examine the resettlement experiences of former refugees living in regional Australia, focusing on mental health and support services, including barriers to access. | Major stressors:  Accessing employment, housing, and learning English.  Past trauma persisted for many people, and in addition, they felt guilty and responsible for their families back home.  Barriers to accessing mental services include:  A lack of English language proficiency and a lack of interpreters (often children play that role).  A lack of understanding of the Australian health system (wait lists, costs, and difficulties accessing appointments).  Treatments that follow a Westernized medical model without taking culture or beliefs into account are to blame for cultural misconceptions.  Trauma-informed care in relation to the cognitive impacts that affected learning ability and the grief caused by family members still living in hostile environments. |
| Smith et al. (2020)  In-kind support by the Centre for Rural Health at the University of Tasmania | Regional Launceston, Tasmania, Australia  Mental health and support services | Former refugees who arrived under the Australian Refugee and Humanitarian Program from Burma, Bhutan, Sierra Leone, Afghanistan, Iran, and Sudan, and essential service providers (n = 31).  Data collected from April to July 2018.  Healthcare services. | (1) Qualitative  (2) Semi-structured interviews.  (3) Thematic analysis using Nvivo software. | Examine the environmental, social, and health-related factors influencing the lived experience of resettlement for former refugees. | The importance of English language proficiency for gaining access to jobs, education, and services, as well as the effects of age, trauma, and mental illness on one's capacity to acquire the language.  Inappropriateness and inflexibility of study materials and programme delivery often clash with familial responsibilities.  Desires to retain the language and cultural traditions associated with the home country.  Accessing employment, education, and suitable housing is thought to be essential for successful resettlement.  Barriers like discrimination and non-transferability of qualifications could be addressed by providing incentives to possible employers, industry-specific English language training, and for students, having them assigned based on aptitude rather than age.  Housing affordability in light of escalating costs, the need to feel safe, and the desire to dwell in a familiar setting.  Health status and difficulty accessing services due to a lack of culturally informed practises and interpreters.  Broader social factors and experiences, including having people from their home countries living nearby, support from community leaders and the community, increase feelings of inclusion and belonging.  Discrimination and racism are exacerbated by stereotypical views. |
| Tomasi et al. (2022)  No external funding | Major cities and regional Australia | Refugees (n =1 180) from Iraq (n = 773) and Afghanistan (n = 407).  Five waves from October 2013 to March 2018.  Healthcare services | (1) Quantitative longitudinal study.  (2) Face-to-face or telephone interviews using a questionnaire based on settlement related measures.  (3) Statistical analysis using SPSS. | Identify the predictors of professional help-seeking for mental health problems among Afghan and Iraqi refugees five years post-resettlement. | Significant differences exist between the two ethnic groups.  Professional help-seeking was higher in Afghan women and the elderly and lower in regional Australians.  The likelihood of seeking assistance was lower among Afghans who had less financial difficulty, did not require interpreting assistance, had a driver's license, and knew how to get to government facilities. While both not having any friends in Australia and having a difficult time adjusting were associated with seeking professional assistance.  Among the Iraqis, those with good English-speaking proficiency were less likely to receive help, whereas being older and having a long-term disability or illness were associated with an increased likelihood of receiving professional help.  Refugees in regional areas with likely PTSD were less likely to seek mental health care. |
| Veginadu et al. (2023)  No external funding | Metropolitan and rural areas in Victoria, Australia. | Exploratory ecological design at statistical area level 2 (SA2) targeting the refugee population.  Public dental services | Principal component analysis of select census-derived socioeconomic variables and the integrated need-accessibility index. | Examine the spatial accessibility of public dental services (PDS) relative to the estimated oral health needs of refugee populations. | Spatial accessibility is higher in metropolitan areas than rural areas for both driving and public transport.  Public transportation accessibility is declining in both urban and rural areas.  Different oral health needs:  In rural areas, 47.6% are in the "high" needs category and 10.7% are in the "low" needs category, compared to metropolitan areas, where 25.4% are in "high" needs and 19.8% are in "low" needs. |
| Willey et al. (2018)  Funded by a small grant from Monash University campus seeding grants programme. | Regional Victoria, Australia | Maternal and child health nurses (n = 26)  May–June 2014  Maternal and child health services | 1) Qualitative descriptive  (2) Community-based participatory research.  (3) Thematic analysis | Explore service provision for Victorian regional refugee families from the perspective of maternal and child health (MCH) nurses. | Nurses were reluctant to ask about the refugee or asylum seeker's background and were lacking information from the referral process. This hindered appropriate care planning.  Culture, family structure, language, and lack of transport impact care continuity and increase the workload for nurses.  Interpreting issues arise with the need to rely on telephone interpreter services or family members.  Limited resources or service policies can make local services difficult to access. Religious groups, playgroups, and multicultural centres were seen as referral options and a means for increasing families’ engagement within the local community. |
| Wood et al. (2019)  No information on funding | Regional NSW, Australia. | Recipients of humanitarian visas, sponsored and supported by a local community organisation for refugees (n = 9).  Country of origin: North-eastern Africa and West Africa  July 2016 to March 2017.  Healthcare services | (1) Qualitative  (2) Semi-structured interviews  (3) Thematic analysis | Explore how employment and volunteering influence the health and wellbeing of refugees and identify areas for appropriate service provision. | Employment or volunteering gave them a strong sense of purpose, which helped them feel worthwhile.  The pressure they felt to succeed as they had to adapt to new workplace cultures and working practises exacerbated uncertainty and anxiety.  Volunteering allowed them to give back, which helped them establish a positive self-image and sped up the process of integrating into the new society.  Employment enabled them to feel valuable rather than like helpless victims and was crucial for men since it allowed them to support their families.  Participants with inadequate English proficiency found it challenging to interact with people outside of their own cultural contexts. This ultimately reduced their confidence and desire to integrate into the larger community and build new lives.  Despite working or volunteering, they continued to face racial discrimination. Employment influences health status overall through both physical activity and the daily routine and purpose it brings. Employment promotes healthy lifestyle behaviours and access to healthcare. |
| Ziersch et al. (2020)  No information on funding | Regional Australia | Online: Medicare Local (ML) (n = 210) and Primary Health Network (PHN) (n = 66) staffs.  Telephone: senior staff, executives, and board council members of MLs (n = 50) and PHNs (n = 55).  Focus groups: (n = 8) with refugee/migrant community groups in each state/territory (n = 62).  Online survey: September and November 2014 and July to October 2016.  Telephone interviews: November 2014 to February 2015, and August and July 2016.  Focus groups: April to June 2015.  Medicare Locals (MLs) and Primary Health Networks (PHNs) | (1) Mixed methods.  (2) Online surveys, telephone interviews, and focus groups.  (3) Simple descriptive statistics analysis using SPSS and thematic analysis using the Nvivo software. | Examines how Australian regional primary health care organisations –Medicare Locals (MLs) and Primary Health Networks (PHNs)—have engaged with migrant and refugee health and what factors encourage work in this area. | From MLs and PHNs:  In 46% of MLs and 74% of PHNs, migrant and refugee health issues were noted in needs assessment documents. However, 78% and 62%, respectively, did not report any activities for refugees.  Activity types in annual reports:  ‘Clinical service provision’ (i.e., screening programs).  ‘Service facilitation’ (i.e., cultural awareness training for GPs to fund interpreter services and transport services).  ‘Health and health system education’ ((i.e., flyers in different languages through to refugees).  ‘Health promotion’ (i.e., mental health awareness in an inclusive community).  ‘Community health program’ (i.e., provision of small grants).  ‘Community engagement/input’ (i.e., consultation with culturally diverse groups).  ‘Policy advice to the states.’  Key factors impacting refugee/migrant health:  Local priority, as migrants and refugees represent only a small proportion of the population.  Policy context and funding.  Collaboration with migrant and refugee organisations and communities, and mechanisms for engagement.  From migrant and refugee organisations:  Language barriers and poor access to interpreters;  Limited numbers of bilingual General Practitioners (GPs) and health workers.  Unaffordable cost and low numbers of GPs bulk billing.  Difficulties navigating the health system.  Broader social needs include housing, social connections, education and employment, and – for refugees in particular – issues of safety.  Collaboration with migrant and refugee organisations and communities and mechanisms for engagement. |
